# Newborn technology use in low-resource settings: the role of health professionals’ communication in implementation

**DOI:** 10.1093/heapol/czaf066

**Published:** 2025-09-12

**Authors:** Gloria Karungo Ngaiza, Dorothy Oluoch, Sassy Molyneux, Caroline Jones, Mike English, Catherine Pope

**Affiliations:** Health Systems Collaborative, Nuffield Department of Medicine, University of Oxford, Peter Medawar Building, 3S Parks Road, Oxford OX1 3SY, United Kingdom; Health Systems and Research Ethics, Kenya Medical Research Institute (KEMRI)-Wellcome Trust Research Programme, Second Floor, 197 Lenana Place, Lenana Road. P.O. Box 43640, Nairobi 00100, Kenya; Health Systems Collaborative, Nuffield Department of Medicine, University of Oxford, Peter Medawar Building, 3S Parks Road, Oxford OX1 3SY, United Kingdom; Health Systems and Research Ethics, Kenya Medical Research Institute (KEMRI)-Wellcome Trust Research Programme, Second Floor, 197 Lenana Place, Lenana Road. P.O. Box 43640, Nairobi 00100, Kenya; Health Systems Collaborative, Nuffield Department of Medicine, University of Oxford, Peter Medawar Building, 3S Parks Road, Oxford OX1 3SY, United Kingdom; Health Systems Collaborative, Nuffield Department of Medicine, University of Oxford, Peter Medawar Building, 3S Parks Road, Oxford OX1 3SY, United Kingdom; Nuffield Department of Primary Healthcare Sciences, University of Oxford, Radcliffe Primary Care Building, Radcliffe Observatory Quarter, Woodstock Road, Oxford OX2 6GG, United Kingdom

**Keywords:** technology, newborn care, health professionals, communication

## Abstract

Neonatal deaths remain a critical public health challenge in many low- and middle-income countries (LMICs), including Kenya. Affordable technologies such as Comprehensive Positive Airway Pressure (CPAP) and phototherapy machines can reduce neonatal mortality and are used in these settings. However, their introduction and implementation in resource-constrained health system contexts are poorly understood. This study investigates how communication among health professionals influences decisions to use CPAP and phototherapy devices in Kenyan newborn units. Using a focused ethnographic approach, we conducted unstructured non-participatory observations, semistructured interviews, and document reviews in two newborn units in level five Kenyan referral hospitals. The study participants were all health professionals working in the newborn units. We gathered data in two phases, 6 months apart, and analyzed the data thematically. Data collection and analysis were informed by The Non-Adoption, Abandonment, Scale-Up, Spread, and Sustainability (NASSS) framework. We found four interconnected contextual factors that influenced health professionals’ communication on the initiation, maintenance, discontinuation, and repair of neonatal technologies. These factors are as follows: First, physical environment, including space availability, newborn unit layout, and the arrangement of cots and incubators. Second, socio-organizational dynamics, such as the team composition, workload, management approach, and workplace culture. Third, technology-specific attributes, particularly the perceived complexity of CPAP and phototherapy’s features and functions. Finally, the wider system encompasses administrative burdens from research and donor-supported programs as well as political, financial, and regulatory factors. Stakeholders, including funders, policymakers, local governments, and health professionals, must recognize that interconnected physical, organizational, technological, and wider contexts shape communication, decision-making, and use of life-saving technologies. A tailored approach that considers these complex realities, rather than a one-size-fits-all approach, should contribute to better integration and sustainability of these technologies, leading to improved outcomes in newborn care.

Key messages

- Continuous positive airway pressure (CPAP) and phototherapy machines significantly improve newborn care and outcomes, but like many health interventions, their successful implementation depends on effective communication among health professionals.
- There is a notable evidence gap on how communication in low-resource, understaffed settings influences decisions regarding the use of CPAP and phototherapy.
- This study provides a comprehensive analysis of how contextual factors shape communication among health professionals in decision-making about initiating, maintaining, stopping these technologies, and managing technology repairs.
- Using CPAP and phototherapy should account for context-specific factors rather than a universal approach to ensure effective communication, which is crucial for technology integration and sustainability.

## Introduction

Neonatal mortality remains a significant global challenge, accounting for over half of all child deaths under the age of five (Li et al. 2021, World Health Organisation 2022). Just over 2.3 million newborn deaths were reported in 2023, the majority in low and middle-income Countries (LMICs) (UNICEF 2023). Despite some improvement over the last decade, Kenya still faces high neonatal mortality rates, with 21 out of every 1000 newborn babies dying within the first month (KDHS 2022). The leading causes of death among newborn babies in Kenya are birth asphyxia, hypothermia (Olack et al. 2021), neonatal sepsis, respiratory distress syndrome (Irimu et al. 2021, Olack et al. 2021), jaundice, and intrapartum-related complications (Irimu et al. 2021).

Technological interventions have been proven effective in reducing newborn mortality (Bhutta et al. 2014, Rosa-Mangeret et al. 2022, Lawn et al. 2023). Continuous Positive Airway Pressure (CPAP) offers respiratory support for babies suffering from respiratory distress syndrome (Ho et al. 2020), while phototherapy can reduce high bilirubin levels in jaundiced babies (Ansong-Assoku et al. 2024). Despite evidence indicating the positive impact of technologies on neonatal outcomes, their use in routine practice in LMICs remains a challenge (Marks et al. 2019 , Amadi et al. 2024).

Effective communication supports the implementation, uptake, and successful utilization of health interventions (Chichirez and Purcărea 2018, McGuier et al. 2024) and is essential for delivering neonatal interventions (World Health Organisation 2020). Previous studies have examined communication related to newborn technologies in LMICs (Aneji et al. 2020, Kinshella et al. 2020, 2022, Nabwera et al. 2020), but have not explored communication in depth. Consequently, we have limited evidence regarding communication in low-resource contexts and its influence on decisions related to technology usage. To address this gap, this study aims to understand how communication among health professionals in low-resource settings shapes the utilization of neonatal technologies. We examine communication at three key decision-making points regarding CPAP and phototherapy use: (i) initiation and discontinuation of technology, (ii) ongoing use of technology, and (iii) technology malfunctions. For each decision-making point, we explore how the newborn unit context shapes communication among health professionals and, consequently, influences decisions related to CPAP and phototherapy.

## Materials and methods

### Study design

**Figure 1.**
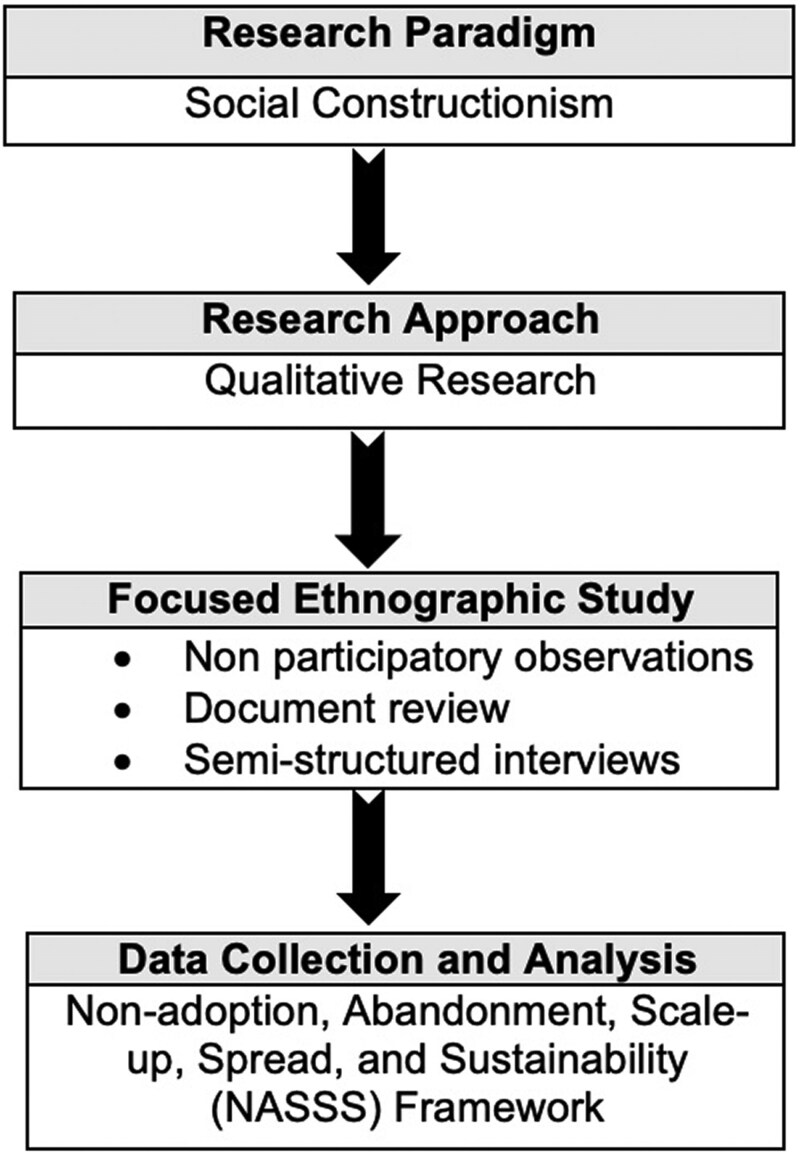
The research approach.

Operating within a social constructionism paradigm that emphasizes the importance of social interactions and shared meanings in shaping knowledge and understanding (Crotty 1998, Burr and Dick 2017), we employed a qualitative, focused ethnographic study design (Trundle and Phillips 2023). Ethnography offers a robust framework for examining cultural practices (Angrosino 2007, Reeves et al. 2008, LeCompte et al. 2010), including the experiences, routines, and interactions of health professionals (Chenhall and Senior 2023). Unlike classic ethnography, which requires extended field immersion, focused ethnography is designed for shorter, more intensive study periods (Müller and Brailovsky 2020). Our data collection methods included non-participatory observations, semi-structured interviews, and document reviews. Figure 1 illustrates the research approach.

The Non-Adoption, Abandonment, Scale-up, Spread, and Sustainability (NASSS) framework (Greenhalgh et al. 2017) guided a significant part of our data collection and analysis, providing a structured framework for examining technology implementation. Derived from 28 technology frameworks and case studies, the NASSS framework has proven particularly valuable for projects designed to improve patient care and outcomes (Greenhalgh et al. 2017, Greenhalgh and Abimbola 2019). As its developers recommended, we flexibly applied the NASSS framework to align with our research focus. As detailed in Table 1, we applied Domain 1 (Condition), Domain 2 (Technology), and Domain 3 (Value Proposition) to examine the attributes of CPAP and phototherapy technologies. Domain 3 was merged with Domain 2, as both refer to the characteristics and perceived value of the technologies. This analysis considered the technology features, functionality, and medical conditions of newborn babies requiring these technologies and how these factors influence communication among health professionals. Domain 4 (Adopters) and Domain 5 (Organization) were used to explore the socio-organizational context of communication about CPAP and phototherapy. The focus was on health professionals’ capacity and capabilities, management styles, and the culture within newborn units. Domain 6 (Wider Context) assessed external factors beyond the newborn unit that shape communication among health professionals. Domain 7 (Embedding and Adaptation Over Time) was treated as a cross-cutting theme across all domains. Additionally, rather than categorizing findings as ‘simple’, ‘complicated’, or ‘complex’ as suggested in the NASSS framework, we focused on broader dynamics within newborn units. We also examined the role of the physical environment in communication among health professionals. This aspect was analyzed separately, as it is not explicitly addressed within the NASSS framework.

**Table 1.** NASSS framework domains operationalized in the study.

	NASSS domains	Study focus
1.	The condition	The medical conditions or illnesses in newborn babies that necessitate the use of CPAP and phototherapy. The severity of these conditions during CPAP and phototherapy use, including any underlying health complications.
2.	Technology	The structure and functionality of CPAP and phototherapy devices. The type and relevance of information generated during the use of CPAP and phototherapy.
3.	Value proposition	The safety and acceptability of using CPAP and phototherapy in the clinical setting. This domain was merged with Domain 2, Technology.
4.	Adopters	Health professionals involved in the use of CPAP and phototherapy, including their skills, routines, and practices.
5.	Organization	The organizational capacity, culture, and management style shaping communication among health professionals in the newborn units.
6.	Wider system	The political, financial and regulatory factors outside the newborn unit shaping communication in using CPAP and phototherapy.
7.	Embedding and adaptation over time	How the technologies are adopted (or not) in everyday practice in the two newborn units and whether this is sustained.

CPAP, continuous positive airway pressure; NASSS, non-adoption, abandonment, scale-up, spread, and sustainability.

### Research settings

The Newborn Essential Solutions and Technologies (NEST360) initiative provided a bundle of newborn care technologies to 13 referral hospitals across Kenya during its first phase of implementation (NEST360 2024). Our research is part of the Learning to Harness Innovation in Global Health for Quality Care (HIGH-Q) study (HIGH-Q 2020). It is a 5-year multidisciplinary program, embedded in four of the 13 county referral hospitals supported by NEST360. These four hospitals were selected in consultation with the Kenyan government and other stakeholders, taking into account factors such as geographical diversity and available funding.

The HIGH-Q study aims to assess the impact of technology adoption in newborn care. The program also examined workforce constraints by adding three additional nurses and ward assistants to each of the study hospitals (Imam et al. 2022). All four hospitals are classified as Level 5 facilities—county referral hospitals that serve as regional hubs for comprehensive inpatient and outpatient care, including medical, surgical, diagnostic, and rehabilitative services (World Bank 2022).

For the specific research detailed in this paper, we collected data from the newborn units of two of the four HIGH-Q hospitals. This choice was driven by the need to conduct detailed ethnographic work, which required extended immersion in the hospital environment. The selection of the two hospitals also aimed to reflect geographical differences and the capacity of the newborn units. Hospital A was situated near Kenya’s capital city and served the largest population among the four sites. It was the busiest, with the highest annual number of neonatal admissions. Conversely, Hospital B was located in a smaller urban center about three hours from the capital. It served the second-smallest catchment population among the four hospitals and had the lowest number of annual newborn admissions.

Approximately 1 year prior to the commencement of the HIGH-Q program, both hospitals received a range of technologies from the NEST360 program and continued to receive support for quality improvement and technology maintenance throughout the study. Hospital A newborn unit had eight CPAP machines and seven phototherapy machines. Three CPAP machines were donated by various sources 5 years before the research, while the NEST360 program supplied the remaining five. Similarly, four phototherapy machines were donated by another source 4 years before the research, and NEST360 provided three newer devices. Hospital B newborn unit had four CPAP machines and five phototherapy machines. The NEST360 program supplied all four CPAP devices and four phototherapy machines, supplementing the unit’s existing single phototherapy machine.

### Recruitment and data collection

Before commencing data collection, we engaged stakeholders at the national, subnational, and facility levels and familiarized ourselves with hospital operations. We established rapport with staff and discussed approaches to minimize disruption during fieldwork. Research activities were conducted in two rounds: the first from November 2021 to April 2022 and the second from October 2022 to February 2023, when three additional nurses were added to each newborn unit as part of a HIGH-Q intervention.

In each round of fieldwork, we collected all data from one hospital before moving on to the next. We first conducted guided non-participatory observations (Supplementary Material S1). Our primary focus was on health professionals, with family members and newborn babies observed only during their interactions with health professionals. Observation periods lasted 9–12 h, alternating between weekdays and weekends and day and night. While doing the observations, we also reviewed documents in the newborn units.

During the final week of each data collection round, we conducted formal, individual, semi-structured interviews with health professionals using an interview guide (Supplementary Material S2). A stratified purposive sampling technique was employed to select health professionals from diverse groups with varying levels of experience and responsibilities. Selection criteria included factors such as age and duration of employment in the newborn units. We conducted interviews until data saturation.

Continuous verbal consent was maintained throughout the study to account for the changing shifts and rotations of health professionals in the newborn units. Additionally, all interview participants provided written informed consent before participating in the study.

Data were gathered by the first author, who is a Tanzanian female doctor and doctoral researcher with approximately 10 years of public health and clinical experience working in hospitals in LMIC settings. She is fluent in Kiswahili and English, the main languages spoken in Kenya. Semi-structured interviews were recorded, transcribed verbatim, and, where necessary, translated into English from Kiswahili.

### Data analysis

Data were analyzed concurrently with data collection, and NVivo 13 was used to support coding and data retrieval. Initial coding was conducted by the first author and subsequently reviewed by the remaining authors. The first author presented proposed codes, which were reviewed by the co-authors across transcripts and field notes in multiple iterative discussions, resulting in a collaboratively agreed-upon coding framework. A combination of deductive and inductive coding was employed. Deductive codes were drawn from the literature on technology adoption, communication, and the NASSS framework domains. Inductive codes emerged from patterns identified directly in the data, including language used by participants and recurring concepts not captured by the reviewed literature. After refining and grouping the codes, we identified four themes. We utilized the One Sheet of Paper method (Ziebland and McPherson 2006) to mind-map ideas and refine our interpretation of the data.

## Results

We conducted 650 hours of observation in each hospital (350 hours during the first data phase and 300 during the second). The document review included patient files, handover books, discharge notes, registers in various areas, and materials posted on the walls. The complete list of the documents we found in the newborn units and reviewed is available in Supplementary Material S3. A total of 42 health professionals were interviewed—19 from Hospital A and 23 from Hospital B, until data saturation was reached. In each hospital, we interviewed one pediatrician and three medical officer interns. Hospital A also included four clinical officer interns, while Hospital B had two in the interviews. Nurses formed the largest group, with nine interviewed in Hospital A and 12 in Hospital B. Additionally, we interviewed two ward assistants in Hospital A and three in Hospital B. Nutrition staff were only available in Hospital B, where one nutritionist and one nutrition intern took part in the interviews.

Table 2 outlines the shift structures and tasks of health professionals in the newborn units of both hospitals. Most health professionals followed a two-shift schedule—day and night. However, in Hospital B newborn unit, nurses worked three shifts. In both newborn units, pediatricians and medical and clinical officer interns also provided care in the general pediatric ward alongside their newborn unit responsibilities. Biomedical engineers and occupational therapists covered all hospital departments, including the newborn units. However, Hospital B had a full-time medical officer in addition to medical and clinical officer interns, whereas Hospital A relied primarily on interns for routine medical tasks.

**Table 2.** Staff roles and tasks in hospitals A and B newborn unit.

		Hospital A newborn unit		Hospital B newborn unit	
Newborn unit capacity		Up to 45 cots and incubators, 40–80 admissions		Up to 30 cots and incubators, 15–30 admissions	
Average annual deliveries		9000 Women delivering		6000 Women delivering	
CPAP and phototherapy		8 CPAP machines, 7 phototherapy machines		4 CPAP machines, 5 phototherapy machines	

Personnel	Parameter(s)	Day time	Nighttime	Day time	Nighttime
	**Shift (s)**	8:00 a.m. to 6:00 p.m.	6:00 p.m. to 8:00 a.m.	Nurses: 7:30 a.m. to 4:30 p.m. and 12:30 to 6:30 p.m. Other health professionals: 7:30 a.m. to 6:30 p.m.	6:30 p.m. to 7:30 a.m.
Nursing team	Number	2–4 Nurses daily 6–10 Students daily	1–2 Nurses daily 2–4 Students daily	2- 3 Nurses daily 4–10 Students daily	1–2 Nurses daily 4–10 students during weekdays
	Tasks	Nursing students: patient medication Nurses: patient medication, handover, monitoring patients.	Nursing students: patient medication Nurses: patient medication, handover, monitoring patients.	Nursing students and nurses: patient medication, handover, monitoring patients.	Nursing students and nurses: patient medication, handover, monitoring patients.
Medical Team	Number	1 Pediatrician, twice a week 2–3 Medical officer interns daily 2–4 Clinical officer interns daily 10–20 Medical students, twice a week	1 Medical officer intern	1 Pediatrician, once a week 2–4 Medical officers, twice a week 2–4 Medical officer interns daily 2–4 Clinical officer interns daily	1 Medical or clinical officer intern
	Tasks	Cannulation, ward round, patient admission and discharge	Cannulation, patient review and admission	Cannulation, ward round, patient admission and discharge	Cannulation and patient review and admission
Nutritionists	Number	2 Nutritionists, three times a week		1 Nutritionist, during weekdays	
	Tasks	Weighing babies		Weighing babies, providing nutritional advice to mothers of sick babies	
Occupational Therapists	Number	1 Occupational therapist, twice a week		1–2 Occupational therapists, during weekdays	
	Tasks	Attending to babies with physical, and developmental challenges		Attending to babies with physical, and developmental challenges	
Biomedical Engineers	Number	1–3 Biomedical engineers, twice a week		3 Biomedical engineers, once a week	
	Tasks	Technology repair, maintenance		Technology repair, maintenance	

CPAP, continuous positive airway pressure.

During the nine weeks of observation at each site, Hospital A had an average of one newborn baby per day using CPAP and five receiving phototherapy. In Hospital B, one newborn baby used CPAP daily, while three received phototherapy. Communication among health professionals in both hospitals occurred through verbal, non-verbal, and written forms. It was more frequent during day shifts, coinciding with higher activity levels and the presence of more health professionals.

**Figure 2.**
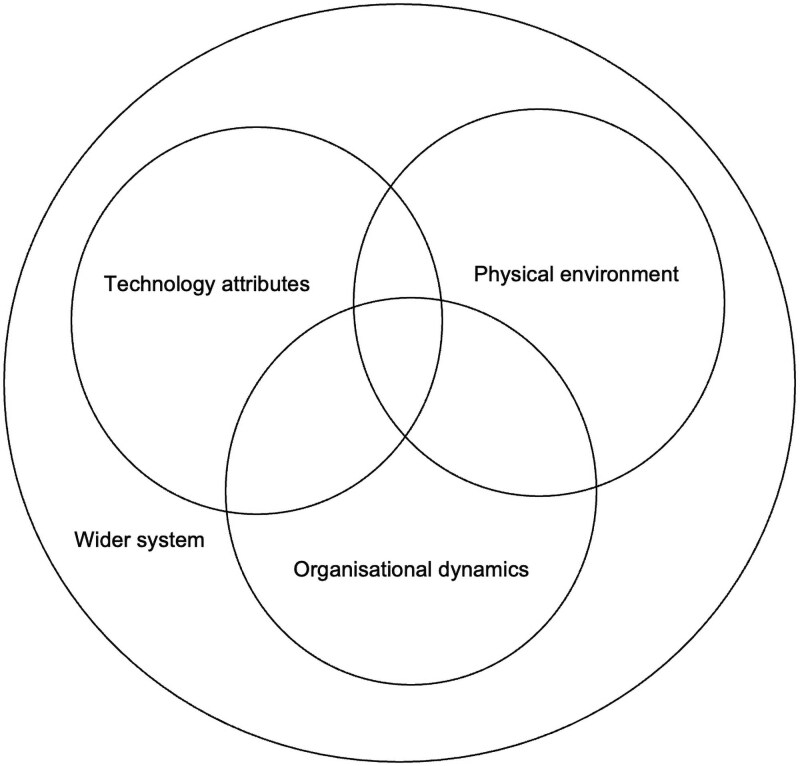
Contextual factors shaping technology-associated communication among health professionals.

As illustrated in Fig. 2, we demonstrate how communication among health professionals in the newborn unit is influenced by four contextual factors: the physical environment, organizational dynamics, technology attributes, and the wider system. Our analysis centered on communication during three critical decision-making points: (i) initiating and discontinuing CPAP and phototherapy, (ii) maintaining and monitoring their use, and (iii) managing technology repairs.

### Physical environment

The physical environment of the newborn units influenced when, how, and to whom information about technology use was communicated. In Hospital A, overcrowding was a constraint, with the unit accommodating up to 80 babies alongside their mothers, who visited every 2–3 hours, and multiple health professionals. The combination of high patient volume, frequent movement, and the presence of medical equipment created a noisy and congested environment. These conditions restricted verbal exchanges and physical movement, limiting opportunities for discussions about initiating or discontinuing CPAP and phototherapy or addressing equipment failures. In contrast, Hospital B newborn unit, though physically smaller, housed a maximum of 30 babies—only 40% of Hospital A’s capacity. This lower patient load and patient proximity allowed for more structured interactions, enabling health professionals to gather and engage in deliberate discussions about treatment decisions, machine settings, and repair needs. Supplementary Material S4 illustrates key differences in the capacity of the two newborn units.

The layout of the units further shaped communication dynamics. In Hospital A, patient rooms were widely spaced, requiring health professionals to walk long distances to exchange information. This physical separation contributed to communication delays, particularly when urgent decisions were needed. In contrast, the Hospital B newborn unit’s layout was designed around a central communal area (health professionals’ bay) for health professionals, facilitating frequent and informal staff exchanges. This proximity facilitated information flow, reducing the risk of miscommunication and delays in making critical decisions, such as adjusting CPAP pressure settings or discontinuing phototherapy.

Another key layout factor was the arrangement of cots and incubators for babies receiving CPAP and phototherapy. In Hospital A, babies using technologies were scattered throughout the newborn unit, increasing the likelihood of being overlooked during routine monitoring and documentation. This fragmented arrangement created information gaps, affecting clinical decisions such as technology-related dosage adjustments and treatment duration. In contrast, the Hospital B newborn unit had a designated area for babies receiving CPAP and phototherapy, ensuring that health professionals could systematically monitor their progress. This spatial organization of the newborn units was observed alongside structured handovers, detailed documentation, and clear decision-making processes, such as those related to oxygen levels for CPAP and jaundice levels for phototherapy.

### Technology attributes

We applied three domains of the NASSS framework—(i) Condition and (ii) Technology (combined with domain (iii) Value Proposition)—to explore how the characteristics of the medical conditions and the attributes of the technologies influence communication patterns among health professionals in the neonatal units.

#### Condition

The nature of the medical condition and its perceived severity played a role in shaping communication regarding the use of CPAP and phototherapy. Jaundice, a condition commonly treated with phototherapy, was frequently regarded as relatively ‘simple’ by health professionals. This perception arose from the generally stable condition of most affected babies and the predictable improvement observed after initiating treatment. As a result, communication about decisions to use phototherapy was often brief, minimal, and perceived as low-risk.

It [jaundice] is something you [health professionals] can make an observation by yourself and make the decision. (Interview, Nurse 1, Round 1, Hospital A)

This simplicity contrasted with the more perceived critical conditions requiring CPAP, such as respiratory distress. Babies in need of CPAP were viewed as being in a more unstable state, prompting more frequent discussions and higher levels of deliberation among health professionals. The perceived complexity and higher risk associated with CPAP use led to more extensive communication, particularly during and after the therapy's initiation.

Because it is just the heaviness of the medical issue, and you find that you cannot compare a jaundiced patient with a patient who is already in meconium aspiration syndrome [one cause of respiratory distress]. The patient who will require the most attention will obviously be meconium aspiration syndrome. (Interview, Medical Officer Intern 1, Round 1, Hospital A)

For those babies born before 32 weeks, at that time the surfactant has not matured. I have to start CPAP before I wait for that baby to start deteriorating. (Interview, Nurse 1, Round 1, Hospital B)

#### Technology

The perceived complication and functionality of the technologies also shaped communication. Phototherapy was regarded as a straightforward technology due to its simple design and low-risk nature. As a result, its use involved less consultation and deliberation among the healthcare team, with decisions made more quickly.

I would not say I have experienced any challenges when it comes to starting babies on phototherapy, and the baby will survive. (Interview, Paediatrician 1, Round 2, Hospital A)

It is something that you can see, and it does not require a lot of connections. So, you can start without much discussion. (Interview, Nurse 8, Round 1, Hospital B)

In contrast, CPAP was described by health professionals as more complicated to use, due to its invasive nature, the presence of multiple tubes, operational noise, and handling difficulties. The need for specialized knowledge and skills for practical use was connected to more frequent consultations among health professionals, often leading to hesitations about initiating or discontinuing treatment. Health professionals in both hospitals reported spending more time monitoring the progress of babies on CPAP, focusing on indicators such as oxygen saturation and respiratory rate, even in cases where they did not strictly follow established guidelines.

Once you start the CPAP, you have to monitor closely. (Interview, Nurse 1, Round 2, Hospital A)

You know, phototherapy, you just put the baby under the light, but for CPAP, it looks as if it is more complicated, like, there needs to be consultations. (Interviews, Medical Officer Intern 3, Round 1, Hospital B)

Frequent malfunctions in pulse oximeters, which are essential tools for monitoring newborn babies’ responses to CPAP treatment, increased the perceived complexity of using CPAP. Health professionals reported, and we observed, that these malfunctions led to extended communication, as they had to report and follow up on repair requests with biomedical engineers.

I think the biggest worry is CPAP. These days, we do not have enough working pulse oximeters to keep the baby on the monitors. So, we just connect the CPAP and we just view it. So, we are not sure. Okay, we can intermittently check the saturations, but ideally, how we were trained, if the baby is on CPAP, you are supposed to be on a monitor so that you can see how the baby is faring. (Interview, Paediatrician 1, Round 1, Hospital A)

All three NEST360 and one other pulse oximeter are not working. We took them to the biotechnicians, and they said the problem is with sensors…. We cannot use CPAP because we need to monitor the oxygen saturation pressure. (Interview, Nurse 4, Round 2, Hospital B)

### Organizational dynamics

We applied Domains 4 (Adopter System) and 5 (Organization) of the NASSS framework to analyze how organizational dynamics shaped communication among health professionals using CPAP and phototherapy.

#### Adopters

The composition and workload of health professionals in the newborn units directly influenced communication. In Hospital A, a high patient-to-staff ratio and role restrictions limited interactions around technology use. For example, clinical officer interns were not permitted to be on call over 12-hour night shifts, increasing the workload for medical officer interns and reducing opportunities for peer discussions, advice-seeking, and feedback on CPAP and phototherapy use. We noted that this dynamic constrained collaborative decision-making and further impacted documentation accuracy as overburdened staff struggled to record essential patient data. Conversely, Hospital B had more health professionals available per patient (although still low) and greater flexibility in role allocation. For example, both clinical and medical officer interns were allowed to be on call at night, and nursing students played a more active role in patient care compared to those at Hospital A. Health professionals reported, and we observed that this distribution of responsibilities allowed for more frequent communication, particularly regarding guideline adherence, collaborative problem solving, and comprehensive documentation.

Access to training also played a critical role in shaping communication. Despite being part of a program prioritizing quality improvement and training, Hospital A lacked a structured mentorship system for new staff in the newborn unit, given the continuous rotation of medical and clinical officer interns. Health professionals reported that this gap created knowledge inconsistencies among health professionals, affecting their confidence in discussing and managing CPAP and phototherapy use.

You may see we may do a CME [Continuous Medical Education] on CPAP, and someone [clinical or medical officer intern] is in their seventh week, another one is in their second week, and, you know? Yeah. So, the level of knowledge is not uniform for all of them. (Interview, Paediatrician 1, Round 1, Hospital A)

In contrast, Hospital B’s organized mentorship structure allowed health professionals more time to engage in skill-building activities beyond formal training. They participated in cross-professional learning sessions, fostering a more profound understanding and confidence in decision-making. We observed that these opportunities facilitated active communication, as staff were more comfortable discussing treatment options, troubleshooting issues, and providing peer support.

Because they [junior doctors] usually rotate, if the new staff come, we usually train them. (Interview, Nurse 3, Round 1, Hospital B)

#### Organization

Organization dynamics shaped communication among health professionals within the newborn units, particularly regarding leadership structures, language use, and accountability mechanisms.

Hospital A operated under a rigid hierarchy in which paediatricians made key decisions. This structure limited the autonomy of other health professionals, restricting their ability to initiate CPAP or phototherapy without explicit approval.

We [nurses] are not allowed to start. Well, there is a consultant who said, during the rounds, that CPAP is supposed to be done by the doctors. So that meant you are not supposed to do it. (Interview, Nurse 1, Round 1, Hospital A)

The hierarchical environment also discouraged interprofessional dialogue, as nurses and clinical officer interns reported feeling hesitant to contribute to discussions about technology use. Many felt undervalued, which negatively impacted their willingness to engage in decision-making.

One case is the fact that you [clinical officer intern] are not allowed to clerk a patient; you are not allowed to make changes on the patient sheet even if you find a mistake……you being a clinical officer intern, they [paediatricians] do not take you as a medic. So, you feel like you are just pushing the ground at times…… We raised them [concerns] to our in-charge, but basically, he does nothing about it, and so at times, you just flow with the system. (Interview, Clinical Officer Intern 2, Round 1, Hospital A)

In contrast, we observed that Hospital B management promoted a collaborative environment. This approach encouraged open communication among professionals, creating a supportive atmosphere where health professionals could freely share information and seek clarification.

I will say that in this ward, everyone is respected. Every decision that you make, as long as it is the best decision. I think there is no conflict about that. So, everyone's decisions matter. (Interview, Nurse 9, Round 1, Hospital B)

The mutual respect culture among health professionals facilitated teamwork by enabling open communication about CPAP and phototherapy, which included a wide range of health professionals and allowed diverse perspectives to inform clinical decisions.

Later, the nurse who came in the afternoon, politely requested the medical officer intern, ‘Please change the antibiotic [Ceftriaxone] because the baby on phototherapy has jaundice and needs a different antibiotic to avoid possible side effects and complications’. (Fieldnotes, Round 2, Hospital B)

The differences in languages spoken by health professionals in Hospital A contributed to communication challenges. English, perceived as formal and hierarchical, was predominantly used by pediatricians, while medical officers, clinical officers, and nurses switched between English and Kiswahili. Other professionals, such as nutritionists and biomedical engineers, primarily spoke Kiswahili. Language differences contributed to the formation of professional silos and limited opportunities for cross-cadre interaction, as health professionals were more likely to communicate within their own linguistic or professional groups. In contrast, health professionals in Hospital B newborn unit mainly used Kiswahili as a shared language among all disciplines, thereby reducing barriers to communication and enabling more consistent information exchange between different health professionals.

Accountability mechanisms also played a crucial role in shaping communication among health professionals about CPAP and phototherapy. In Hospital A newborn unit, limited collaboration between professional groups and low nurse-to-baby ratios contributed to unclear responsibility for documentation and adherence to clinical guidelines. We observed that the absence of structured accountability systems led to inconsistencies in patient records, reduced trust in the data and limited its usefulness for decision-making. Observations revealed instances where health professionals recorded inaccurate information.

The male nurse sat at the nurses’ table, filling out information about the patient’s progress, such as temperature, pulse rate and jaundice status, by making up figures without taking patient measurements…… I later reviewed two files of babies on phototherapy. Nurses’ notes indicated that the patient had no jaundice in the past two days by documenting ‘No jaundice’ in the ‘Jaundice Status’ section of the Comprehensive Newborn Monitoring Chart. However, the medical team notes recorded severe jaundice in the same two days. (Fieldnotes, Round 2, Hospital A)

Hospital B newborn unit, on the other hand, implemented measures aimed at strengthening accountability. Documentation responsibilities were distributed across the team, contributing to the reliability of patient records. Health professionals reported being motivated to maintain accurate documentation, knowing their colleagues depended on the data for clinical decision-making. Cross-professional data-sharing further strengthened this accountability, as staff was aware that incomplete or incorrect records would be scrutinized.

What we did when we found that we are not having so many babies who were being initiated on CPAP, we introduced a book where we will be recording the baby we put on CPAP when you started and when the baby was weaned out of CPAP. So, in that book, we wanted to know who the champion was. So, it has really helped us when we started recording those babies we are putting on CPAP and who have put them on CPAP. (Interview, Nurse in charge, Round 2, Hospital B)

Additionally, both newborn units benefited from informal support structures that facilitated communication. Networks of health professionals provided support and fostered a collaborative working environment. The presence of technology champions—individuals who actively promoted CPAP and phototherapy use—encouraged open discussions and improved confidence in technology management.

I like to get advice and learn from them [other medical team members] because sometimes what is written in the books is not practical, particularly in a limited resource setting. They can advise on when to start a certain therapy, for example, oxygen or CPAP. (Interview, Medical Officer Intern 5, Round 1, Hospital A)

I was not trained in CPAP, but my fellow nurse [the champion] mentored me. (Fieldnotes, Nurse 2, Round 2, Hospital B)

### The wider system

Although our research focused on the newborn unit, broader contextual factors, including political, regulatory and financial aspects, emerged as critical influences on communication regarding CPAP and phototherapy.

At the national level, the Kenyan Ministry of Health, in collaboration with stakeholders, developed and disseminated standardized protocols and guidelines for CPAP and phototherapy to promote consistency across health facilities. Both newborn units in our study were located in counties where political administrations endorsed these technologies.

During its implementation, the NEST360 program provided financial support to both newborn units for specific activities, including training health professionals, technology maintenance, and data use. However, other operational costs depended on county-level funding, the Linda Mama insurance scheme (a nationwide insurance scheme designed for maternal care), and contributions from families of admitted newborns. The extent of financial coverage varied between facilities, directly affecting communication regarding CPAP and phototherapy use.

In Hospital A, families bore the direct costs of CPAP and phototherapy since the Linda Mama insurance scheme did not extend to neonatal care. This financial burden affected communication around the use of these technologies. For example, due to cost concerns, health professionals sometimes omitted essential investigations, such as bilirubin testing, necessary for evidence-based communication regarding initiating or continuing phototherapy.

For blood investigations, their families pay in advance. Most families cannot pay, for example, for bilirubin levels. We just have to start babies on a phototherapy machine before checking bilirubin levels, contrary to the guidelines. (Interview, Nurse 5, Round 1, Hospital A)

In contrast, Hospital B benefited from extended Linda Mama coverage that included newborn babies, supplemented by county government support. This financial stability allowed health professionals to conduct necessary investigations in a timely manner, facilitating more transparent communication and better adherence to national guidelines.

Despite these financial mechanisms, health professionals in both facilities expressed concerns about inadequate county-level funding to hospitals, which undermined essential activities such as paying for staffing, continuous training and orientation, and ensuring monitoring and documentation materials are available for effective communication. The uncertainty about CPAP’s long-term availability led to health professionals reporting hesitancy to invest time in a technology that might become unavailable long term. Logistical challenges, such as delays in equipment repairs, diverted time and attention away from patient-focused communication.

We have monitoring machines which should help the babies on CPAP, but the probe is not working. We have asked for a replacement from the hospital administration, but this will take a long time to be fixed. (Interview, Nurse in charge, Round 2, Hospital A)

When the equipment gets broken down, it takes a lot of time to be replaced or repaired. (Interview, Nurse 10, Round 2, Hospital B)

Another wider systemic factor affecting communication was the administrative burden imposed by research and donor-supported program activities within the newborn units. While these initiatives aimed to improve care, they required health professionals to attend meetings, document activities, and participate in interviews and focus group discussions. Health professionals reported that these responsibilities increased workload and reduced the time available for effective communication and decision-making regarding CPAP and phototherapy use. Hospital A newborn unit managed six concurrent projects, while Hospital B handled four, intensifying pressures on clinical care and communication.

Both newborn units also functioned as referral centers for other hospital departments, including labor, postnatal, and gynecology wards, as they were the only units equipped with CPAP and phototherapy technologies for newborn care. Health professionals frequently engaged in seeking information and discussions with referring departments to gather clinical information and clarify situations about the referred babies, diverting their attention from intra-unit communication about newborn management.

During the ward round, the pediatrician looked frustrated by the lack of information about the admitted baby. She asked the medical officer intern, ‘Who admitted this baby, and who accepted the admission? This baby does not need any machine [CPAP] and is stable. ……. Why is a medical officer [in the labour ward] deciding to send a patient to a newborn unit without consultation? They did not even call us’. (Fieldnotes, Round 1, Hospital A)

A nurse from the post-natal ward entered the newborn unit with a file. She spoke to the deputy nurse in charge, sitting at the health professionals’ bay, to get advice on whether the baby should be shifted to the newborn unit for phototherapy. (Fieldnotes, Round 2, Hospital B)

As referral hospitals, both newborn units received frequent consultation calls from lower-level health facilities without CPAP and phototherapy equipment. These calls often involved inquiries about eligibility criteria and appropriate referral timing. While health professionals acknowledged that these consultations expanded access to neonatal technologies, we observed that they also placed additional demands on health professionals, limiting the time for in-depth discussions within the newborn units.

The phone in the nurse’s room rang, and the nurse went to pick it up…… When he hung up, he told me, ‘A lower-level facility wanted to refer a baby for CPAP, but I told them the ward was full and they should try another level 5 hospital, which is nearby’. (Fieldnotes, Round 2, Hospital A)

After placing baby N, who arrived from a lower-level health facility in a cot for phototherapy, the newborn unit nurse told the nurse who brought the baby: ‘Next time you come without filling the baby’s weight [in the referral form], we will make you weigh the baby’. (Fieldnotes, Round 1, Hospital B)

## Discussion

This study provides insights into how health professionals communicate when using CPAP and phototherapy in settings with limited staffing, highlighting the crucial role of effective communication in supporting the successful implementation of newborn care interventions.

Our findings demonstrate that communication dynamics are shaped by physical, organizational, technological, and broader systemic factors, which, in turn, influence decision-making regarding the use of these technologies.

Methodologically, this study contributes to the growing body of research on technology integration in newborn care. To our knowledge, it is the first focused ethnographic study in LMICs to comprehensively examine technology-related communication and its impact on care decision-making. By addressing a recognized qualitative evidence gap in health policy and systems research, we underscore the value of ethnographic inquiry in uncovering the social dimensions of technology use. Our application of the NASSS framework represents one of its first empirical uses in LMICs. While the framework provided a valuable lens for understanding technology integration, we identified additional influential factors, such as the physical environment, which are not explicitly included in NASSS.

Our findings reveal that the physical environment—encompassing unit space, layout, and design—is crucial in shaping communication among health professionals, affecting CPAP and phototherapy use. These results align with existing studies that, while not explicitly focused on technology, illustrate how nursing unit design influences communication and decision-making. For instance, research in United States hospitals has shown that cross-shaped decentralized units hinder communication compared to centralized nurses’ workstations (Brewer et al. 2018). Similarly, single-family room designs in Neonatal Intensive Care Units hindered visibility and increased walking distances, complicating communication and monitoring. In contrast, open-bay Newborn Intensive Care Unit layouts facilitated interaction and monitoring (Shahheidari and Homer 2012, Doede et al. 2017). Despite this evidence, specific design requirements for optimal communication during the use of newborn technologies like CPAP and phototherapy in low-resource settings have received little attention (Amadi et al. 2023).

Organizational dynamics interplayed with the physical environment to influence communication, with high workloads clearly a significant barrier, primarily due to under-staffing even after adding three nurses in the two newborn units. We emphasize the temporal constraints health professionals face, noting that increased time pressure exacerbates communication challenges—mainly when dealing with complex technologies used in critically ill neonates. Our findings are consistent with studies from Malawi, where low staffing levels led to reduced CPAP use and irregular phototherapy monitoring (Kinshella et al. 2020, 2022). Similarly, high staff turnover and strikes in Kenya compromised communication and documentation for babies receiving CPAP treatment (Nabwera et al. 2020). Beyond newborn technology, high workload has been linked to increased adverse events in Brazil (Lamy Filho et al. 2011), missed nursing care in the United States (Tubbs-Cooley et al. 2019), and heightened stress during neonatal resuscitation in Canada (Zehnder et al. 2020).

Further organizational dynamics, including the newborn unit culture, shaped health professionals’ communication and, as a result, their decisions to engage with CPAP and phototherapy. Hospital A newborn unit’s rigid hierarchy and weak accountability mechanisms hindered effective interaction. In contrast, the collaborative culture, accountability systems, and the supplementary role of nursing students in Hospital B newborn unit fostered open communication, leading to better decision-making and improved patient care. Some of these findings echo studies from Malawian hospitals, where hierarchical structures and management styles meant clinicians were the primary decision-makers regarding CPAP use, even though trained nurses could initiate CPAP during critical situations (Kinshella et al. 2020; Nyondo-Mipando et al. 2020). The language spoken by health professionals played a crucial role in shaping organizational dynamics, fostering teamwork when a common language was shared or exacerbating hierarchy when language barriers existed among different groups. While studies have discussed the impact of language barriers between health professionals and patients (Meuter et al. 2015, Ali and Watson 2018, Srivastava 2019, Al Shamsi et al. 2020, Albury et al. 2020, Pandey et al. 2021, Caitríona and Zoë 2022), there is limited evidence on the role of language used by health professionals in their communication. Our findings highlight the importance of considering the role of language in technology implementation, particularly in contexts where health professionals speak multiple languages.

Interacting with the physical environment and organizational dynamics to influence communication were several technology attributes influencing communication about CPAP and phototherapy. By conducting the study in two hospitals, we were able to isolate technology-specific attributes from contextual factors. Conditions viewed as less severe, such as neonatal jaundice needing phototherapy, resulted in more straightforward communication, whereas complicated conditions necessitating CPAP prompted more frequent and deliberative exchanges among health professionals. Similarly, phototherapy, seen as a simpler technology, required minimal discussion, while CPAP’s complexity necessitated more intensive communication, particularly regarding equipment monitoring and troubleshooting. This finding contributes to the scarce literature on this topic, aligning with a study in India, which indicates that health professionals were more likely to initiate CPAP rather than ventilators due to the perceived simplicity of CPAP (Dewez et al. 2018). Similarly, positive perceptions of oxygen therapy in Australia were linked to its perceived safety (Peeler et al. 2015).

Beyond the newborn unit, we identified various wider systemic factors that shaped communication among health professionals using CPAP and phototherapy machines. While national policies provided structured guidelines for their use, effective implementation depended on factors such as healthcare financing, workforce capacity, and competing clinical priorities. Previous research, such as a study on phototherapy use in four hospitals in Malawi, similarly found that resource shortages—such as the lack of bilirubin tests—negatively influenced health professionals’ communication and decision-making regarding phototherapy machine use (Kinshella et al. 2022). Another critical systemic factor was the coordination of multiple programs within newborn units to reduce fragmentation and duplication among health professionals and free up time for communication regarding technology use. Additionally, challenges within the referral system between departments and hospitals contributed to coordination inefficiencies affecting communication about CPAP and phototherapy. A scoping review of factors influencing the successful implementation of referral systems highlights the importance of well-planned and coordinated processes (Seyed-Nezhad et al. 2021). Similarly, a study on referral challenges in Kenya (Nguru and Ireri 2022) underscores the significance of functional coordination and feedback mechanisms in supporting patient management within complex healthcare environments.

These four intersecting factors are integral to optimizing communication and facilitating effective decision-making. To ensure the successful integration of newborn technologies, stakeholders—including development partners and international organizations—must adopt flexible, context-specific strategies tailored to individual health facilities. Key approaches include optimizing newborn unit design, ensuring adequate staffing, fostering a collaborative organizational culture, strengthening mentorship programs, providing clear clinical guidance, and securing sustained financial and material resources. Furthermore, stakeholders should consider the implementation context of medical technologies like CPAP and phototherapy—often designed for high-income settings—to ensure their smooth integration and long-term sustainability in LMICs. In the Kenyan context, our findings can inform ongoing initiatives, such as integrating technology-specific elements into communication training programs (Musitia et al. 2022) and health service delivery redesign (Croke et al. 2022) initiatives focused on enhancing outcomes, equity, and quality.

This study has several limitations. First, it was conducted in two county referral hospitals, whose contexts may differ from private, faith-based, or lower-level facilities. Second, the study concentrated on CPAP and phototherapy; findings may vary when engaging with other technologies. Third, data collection occurred after introducing these technologies, limiting insights into pre-implementation experiences. Finally, the dual role of the researcher—as both a data collector and a medical doctor—provided valuable clinical insights but posed ethical challenges, particularly in responding to missed care or medical emergencies. Research conducted in newborn units in Kenya has highlighted similar challenges in data collection, including researchers being asked to assist in clinical activities and the impact of research participation on healthcare providers’ time (Jepkosgei et al. 2019). To address these issues, our team held debrief meetings to share experiences and establish strategies for handling challenges in the field. Additionally, we provided feedback to study hospitals on ethical concerns that arose during data collection. Despite these limitations, our study advances the understanding of communication in neonatal technology use, offering critical insights for improving health system responses and optimizing newborn care in LMICs.

## Conclusion

This research highlights critical gaps in implementing newborn technologies in under-staffed, low-resource settings by examining how contextual factors shape communication among health professionals. Our findings reveal the complex interplay between physical, organizational, technological, and broader systemic influences, demonstrating how these interconnected factors affect communication and, in turn, decision-making at every stage of technology use—from initiation and maintenance to discontinuation and repair.

To ensure the effective and sustainable integration of newborn technologies, stakeholders at all levels—from international organizations to local health facilities—must move beyond technical solutions and actively consider these contextual realities. Integrating these factors into planning and implementation strategies will strengthen communication and decision-making among health professionals and improve the long-term impact of neonatal care interventions.

## Supplementary Material

czaf066_Supplementary_Data

## Data Availability

The data underlying this article will be shared on reasonable request to the corresponding author.

## Reflexivity statement

This research was conducted by a multidisciplinary team comprising five women and one man, based in high-income (United Kingdom) and low-resource countries (Tanzania and Kenya). G.N., a Tanzanian medical doctor, led the study as part of her PhD at the University of Oxford. D.O., a Kenyan social scientist at KEMRI Wellcome Trust Research Program, has over a decade of experience researching maternal, neonatal, and child health, health systems, and patient experiences in Kenya. S.M., C.P., M.E., and C.J. are British professors at the University of Oxford. S.M. researches health policy, systems research, and empirical ethics, bringing over 20 years of experience in Kenya. C.P. is a professor of medical sociology and an expert in qualitative research methods, having studied healthcare organization, service delivery, workforce issues, and digital health in the United Kingdom. M.E., a pediatrician and health service researcher, has dedicated more than a decade to improving child and newborn care in African district hospitals (including Kenya) and addresses broader health systems issues that affect the quality of care. C.J. has been researching public health interventions using ethnographic methods and has spent over 10 years in Kenya. The team’s diverse expertise and research experience enriched the study, providing valuable perspectives.
