# Supplementary material for: Newborn technology use in low-resource settings: the role of health professionals’ communication in implementation
Source: Health Policy Plan. 2025 Sep 12;40(10):1056–68. doi: 10.1093/heapol/czaf066 (PMC12605805; doi:10.1093/heapol/czaf066)
Supplement: czaf066_Supplementary_Data [file czaf066_supplementary_data.docx]

**SUPPLEMENTARY MATERIALS**

**Supplementary Material 1: Observation Guide**

The research team will observe the following:

1: Team composition

- Diversity and team composition? (Age, gender, cadres, hierarchy levels)
- What role does each team member play?
- How are tasks shared amongst staff?

2: Context

- Observe resources available
- What are the challenges faced by staff in relation to resources and technologies, and how does this affect their work?
- How do staff deal with these challenges, and what adoptions or changes do they make to make things work with the available resources?
- How do staff interact with families/mothers? Is there task sharing, and how is this supported? Are mothers/families provided with information about the new technologies?
- What do mothers/families feel or say about the new technologies?

3. Individual competencies

- How are individual skills and abilities demonstrated during the use of the technologies?
- Is there training and support for the staff and mothers/families

4. Team interactions with technology

- Observe the process of how new technologies are introduced (by who, who is involved, is there a training/orientation)
- What do staff say about the new technologies (how do they talk about it, what they say about it, ease of use)

Do team members make an effort to participate in the discussions, or are they called upon, encouraged by the team leader?

**Supplementary Material 2: Health Professionals Interview Guide**

Participant Name: _______________________________________________________

| **Signature of researcher:** |  | **Date:** |  | |
| --- | --- | --- | --- | --- |
|  |  | | | |
|  |  |  | |  |
| **Researcher name:** |  | **Time:** | |  |
|  |  |  | |  |
|  | ***(please print name)*** |  | | |

*Probe: Medical care? Nursing care? What are the most important practices? What about the way people work – on the ward, within the hospital? What about relationships with mothers? Do you think everyone shares your views?*

1. There have been several interventions to improve care by the NEST360 team, which of these interventions were useful in improving care (and which did not result to care improvement)? Why do you think they led to care improving / not improving – for who and for what reason?

*Probe: Do you think other healthcare providers found these interventions led care improvement? Who has taken the lead in trying to introduce/champion interventions? Can you tell me a bit about how these interventions led care improvement?*

1. Do you think the senior staff [Hospital administration / Senior Hospital personnel] have found these interventions useful and consider them as one way of improving care for small and sick newborns? Have they supported any of the interventions? Might you have an example?

*Probe: Are you happy with the support you are receiving from senior management? Do you think their view of newborn care provision has changed - Why / Why not?*

1. Are you aware of the performance of your newborn care team in the provision of newborn care services and implementing interventions for small and sick newborns? Have you seen any feedback reports or had feedback presented/discussed with you – in what ways? Do you think the feedback is helpful to you – what is good/bad about it?

*Probe: Do you think the feedback is accurate and fair? Does it result in any real action – can you give an example? Does it help to know how your performance compares to other hospitals?*

1. What do you think were the most critical elements [list NEST360 interventions or activities NEST360 directly influenced] to effecting change in your unit on the NEWBORN UNIT? What were the most exciting things to change? Does the NEWBORN UNIT team have the capacity to change some of the things that are not working well – why / why not?

*Probe: In what ways did the care elements change – can you give an example? Why have some of the things stayed the same?*

1. Do you think are some of the NEST360 interventions that have resulted in any undesirable/unintended effects? Can you give an example? How has this influenced care provision within the NEWBORN UNIT?

*Probe: How did the intervention lead to the undesirable/unintended effect? Do you think this might have been avoided? Has it affected how other interventions are implemented?*

1. Given the opportunity, would you implement the NEST360 interventions differently? Why/why not?

*Probe: What worked well/not well? What minimum resources would have needed to be in place? What additional support would have been useful?*

Thank the interviewee for their time and inform them that they will be made aware of the findings.

**Supplementary Material 3: A list of Documents Reviewed in this Research**

| **DOCUMENTS IN THE PATIENT’S FILE** | | | | |
| --- | --- | --- | --- | --- |
| **DOCUMENT** | **HOSPITAL A NEWBORN UNIT** | **HOSPITAL B NEWBORN UNIT** | **REQUIRED DOCUMENTATION FREQUENCY** | **EXPLANATION** |
| **Continuation Form** | 🗸 | 🗸 | Once a day | Documented information from the medical team regarding the newborn babies’ progress and management plans. |
| **Newborn Admission Records** | 🗸 | 🗸 | During admission to the newborn unit | Recorded information about all newborn babies on admission to newborn unit, including their condition on delivery, weight, temperature and skin colour. |
| **Comprehensive Newborn Monitoring Chart** | 🗸 | 🗸 | Every eight hours | Recorded information about newborn babies' progress such as the temperature, heart rate, breathing rate, and oxygen saturation pressure. It also recorded CPAP and phototherapy-related information such as the time of starting and progress. |
| **Nursing Cards** | 🗸 | X | Once per shift and during an emergency in Hospital A newborn unit  12 times per day in Hospital B newborn unit | Documented the management nurses gave a newborn baby from admission to discharge such as medical procedures, medication and calling the doctor to review. |
| **CPAP Monitoring Sheet** | 🗸 | X | Every six hours | Recorded the progress of newborn babies on CPAP, including the machine's status. |
| **Neobac** | 🗸 | X | Before each test | Documented information about newborn babies who needed blood culture and sensitivity tests. |
| **Lab Requisition Form** | X | 🗸 | Before each test | Documented information about newborn babies who needed other laboratory tests apart from blood culture and sensitivity. |
| **Paediatric Treatment Chart** | 🗸 | 🗸 | Two to three times per day | Recorded information about the treatment newborn babies receive while in the newborn unit. |
| **Referral Forms** | 🗸 | 🗸 | During every referral | Documented information about newborn babies who are referred from other facilities such as their name, date of admission, date of referral, referring facility, the reason for referral and the referring clinician. |
| **Paediatric Referral Form** | X | 🗸 | During every referral | Recorded facility-level details of newborn babies who were referred, including the condition at referral and interventions given. |
| **Radiology Request Form** | 🗸 | 🗸 | Before each investigation | Documented information about newborn babies who needed radiology services. |
| **OTHER DOCUMENTS OUTSIDE THE PATIENT’S FILE** | | | | |
| **The Admission Book** | 🗸 | 🗸 | During admission of newborn babies from other departments within the same hospital. | Recorded information about the newborn babies received in the newborn unit, including mother's name and condition, admission reason, newborn baby's weight, mode of delivery and diagnosis. |
| **The Nurse Report Book** | 🗸 | 🗸 | Two times per day in Hospital A newborn unit  Three times per day in Hospital B newborn unit | Documented information useful for nurses’ handovers and briefing doctors from other departments who were requested to review some newborn babies. It included the newborn baby's mother's name, incubator/cot number, days in the newborn unit, diagnosis, and management. |
| **The Report Book for the Matron** | 🗸 | X | Twice per day | Included information about new admissions and very sick newborn babies. |
| **Patient Referral Book** | 🗸 | 🗸 | During every referral | Recorded information about newborn babies who arrived in the newborn unit from other hospitals including the date of birth, time, the reason for referral, date of birth, Human Immunodeficiency Virus (HIV) status, and who received the newborn baby. |
| **The Unpleasant Event Book/ Patient Report Book** | 🗸 | 🗸 | Two to three times per day | Documented unpleasant events such as birth asphyxia and severe respiratory distress. |
| **CPAP Book** | 🗸 | 🗸 | During every start of CPAP treatment | Documented information regarding newborn babies on CPAP, including their date of birth, birth weight, the day started CPAP, the day completed CPAP, gestation age, and the outcome. |
| **Death Book** | 🗸 | X | After every death | Recorded information about newborn babies who died in the newborn unit, including the cause of death and number of admission days. |
| **Duties Book** | 🗸 | 🗸 | Once per week | Documented nurses’ rota. |
| **Random Blood Sugar (RBS) Book** | 🗸 | 🗸 | After every investigation | For writing information about newborn babies who were tested sugar levels, including the time, sugar level and the number of glucose sticks used when doing the test. |
| **Continuous Medical Education** | 🗸 | X | Once per week | Included a summary of topics nurses choose to present to their colleagues and students. |
| **Round book for major ward round** | X | 🗸 | Once per week | Documented information about the medical team decisions during the major ward round/grand rounds including the diagnosis, and the management plan. |
| **Temperature monitoring book** | X | 🗸 | Two to three times per day | Contained information about the temperature of every section of the newborn unit and indicated whether it was adequate or not. |
| **Nutrition support** | X | 🗸 | Once per day | Recorded information about the nutritional support newborn babies receive from the nutritionist and the medical team, including the feeding mode (breastfeeding, expressed or artificial milk), the amount and number of feeds per day. |
| **Equipment documentation book** | X | 🗸 | Once per day | Reported information about the number and condition of medical equipment in the ward. |
| **Non-pharmaceutical commodities book** | X | 🗸 | Once per week | Reported information about the medical supplies, including the date received, quantity used, reference number and the signature of the responsible person. |

| **DOCUMENTS ON THE WALL** | |
| --- | --- |
| **Hospital A Newborn unit** | **Hospital B Newborn unit** |
| Handwritten paper with numbers of discharged mothers who had premature newborn babies and were invited to the hospital for the Premature World Day. | Actions for danger signs. |
| Staff contact numbers – Medical officer interns on call, consultants on call, and various departments in the hospital such as nutrition, counselling, surgery, and mortuary. | A sample of how to fill in the newborn monitoring sheet. |
| National guideline charts on examining a newborn baby, history taking, management of hypoglycaemia, management of exposed newborn babies and screening for retinopathy of prematurity. | Newborn babies monitoring information sheet. |
| Quality improvement dashboard (July to September 2021). | Duty allocation for students. |
| The medical and clinical officer interns’ rota. | Duty rota for morticians. |
| Drug advertisement for Aptamil (Infant and toddler milk formula). | Biomedical engineer contacts. |
| The Dose of Anti-Retroviral (ARVs) drugs for exposed newborn babies. | How to make 500 mls of 10% dextrose. |
| Letters from the management regarding the supervision of students, the protocol for documenting the management of patients, and research fees for students who have placement in the newborn unit. | Two posters – one says to stop and sanitise hands before and after holding a newborn baby, and the second on five critical moments of handwashing. |
|  | Nursing diagnosis /care plans that included respiratory distress syndrome diagnosis, nursing interventions and goals. |
|  | Firinstrome score for assessment of newborn babies. |
|  | A guideline for Nevirapine dose for babies exposed to HIV infection. |
|  | Top 10 conditions of newborn babies admitted at the newborn unit, according to the second quarter of 2021. |
|  | Criteria for discharging newborn babies from the newborn unit. |
|  | A guide on segregation of medical wastes. |

**Supplementary Material 4**


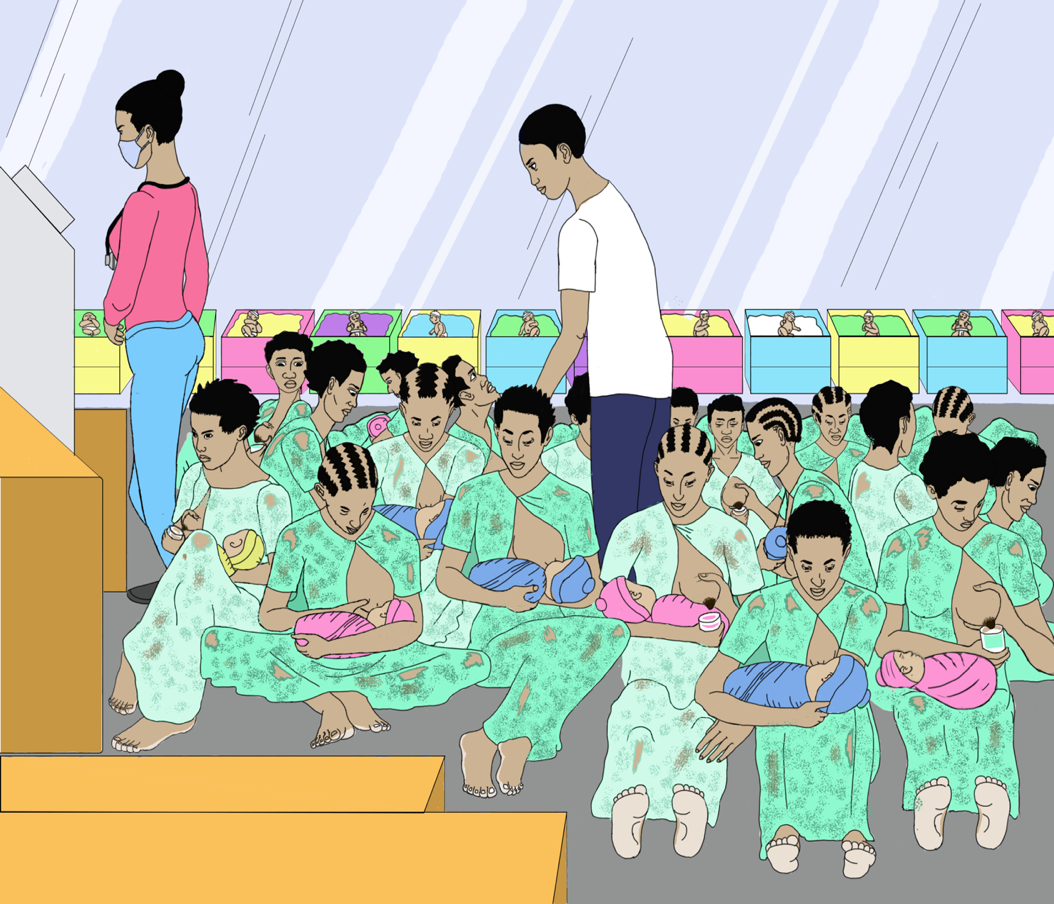


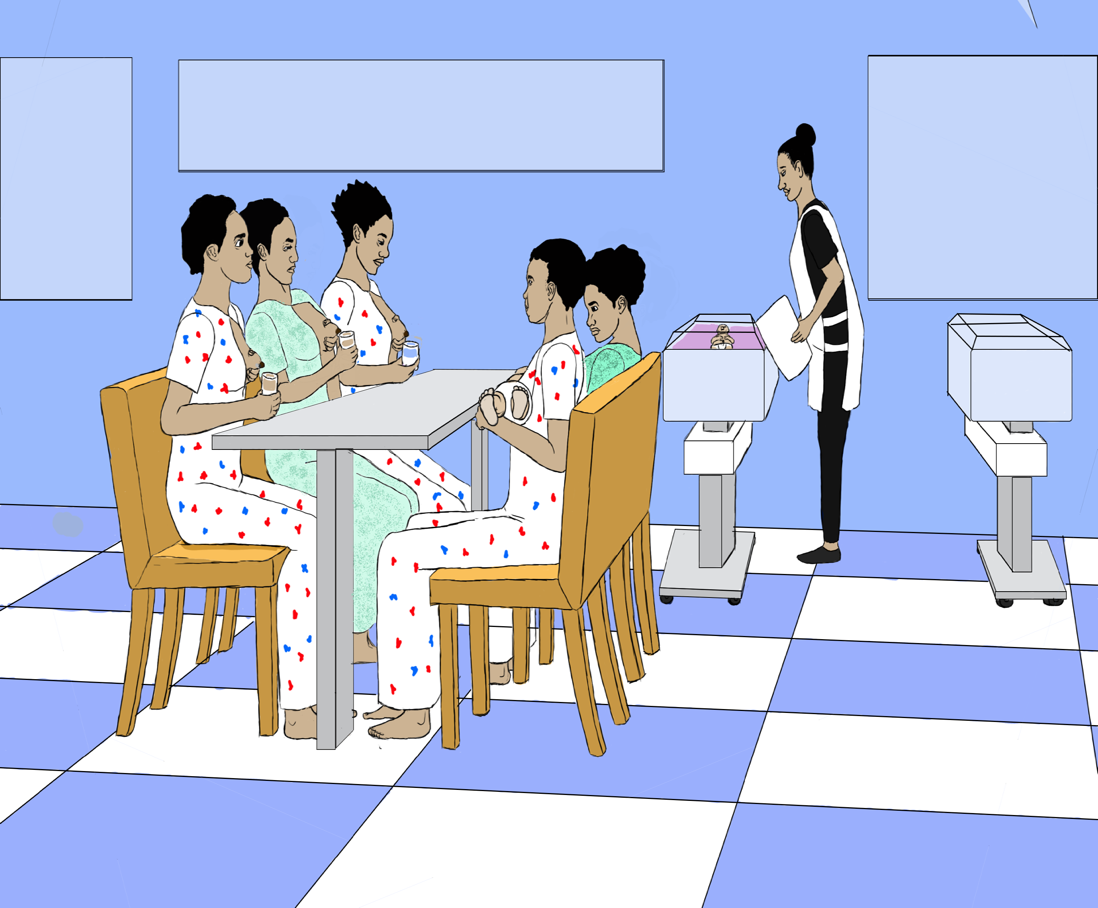


Hospital A Newborn unit Hospital B Newborn unit
